# Supplementary material for: Context value updating and multidimensional neuronal encoding in the retrosplenial cortex
Source: Nat Commun. 2021 Oct 18;12:6045. doi: 10.1038/s41467-021-26301-z (PMC8523535; doi:10.1038/s41467-021-26301-z)
Supplement: Supplementary file 1 — Supplementary Information [file 41467_2021_26301_MOESM1_ESM.pdf]

Supplementary Information

Context Value Updating and Multidimensional Neuronal Encoding in the Retrosplenial Cortex

Weilun Sun[1, 2], Ilseob Choi[1,2], Stoyan Stoyanov[1], Oleg Senkov[1], Evgeni Ponimaskin[3], York Winter[4], Janelle M.P. Pakan[2, 5, 6*], and Alexander Dityatev[1, 2, 7 *]

[1]*Molecular Neuroplasticity, German Center for Neurodegenerative Diseases (DZNE), Magdeburg, Germany*

[2]*Center for Behavioral Brain Sciences (CBBS), Magdeburg, Germany*

[3]*Department of Cellular Neurophysiology, Hannover Medical School, Hannover, Germany*

[4]*Institute for Biology, Humboldt University, Berlin, Germany*

[5]*Institute of Cognitive Neurology and Dementia Research, Otto-von-Guericke University, Magdeburg, Germany*

[6]*German Center for Neurodegenerative Diseases (DZNE), Magdeburg, Germany*

[7]*Medical Faculty, Otto-von-Guericke University, Magdeburg, Germany*

*Correspondence:

Dr. Janelle Pakan
Institute of Cognitive Neurology and Dementia Research,
Otto-von-Guericke University,
Leipziger Str. 44, Haus 64
39120, Magdeburg, Germany
janelle.pakan@med.ovgu.de

Dr. Alexander Dityatev,
German Center for Neurodegenerative Diseases (DZNE)
Leipziger Str. 44, Haus 64
39120, Magdeburg, Germany
Alexander.Dityatev@dzne.de

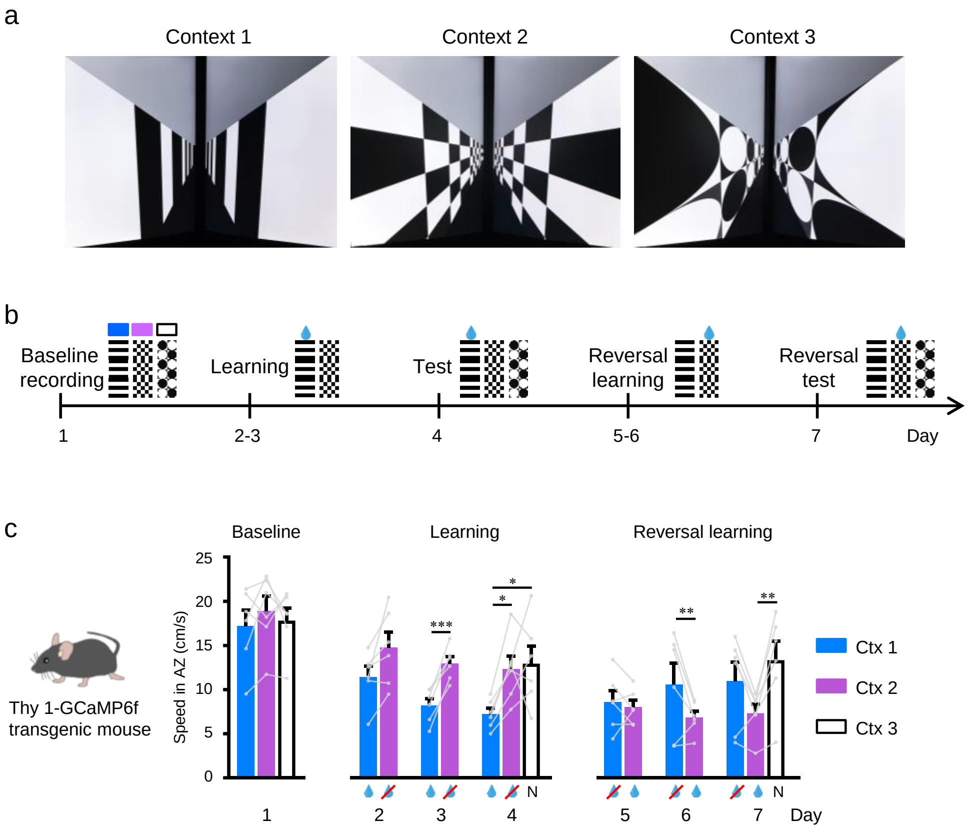

Supplementary Figure 1. Mice learn to associate a water reward with a particular context at a specific position. **a** Snapshots of the presented contexts in the virtual environment. **b** Experimental timeline for control group in which no AAV was injected. Contexts 1-3 (Ctx 1-3) are indicated by their visual patterns and associated color legend. Rewarded context is indicated by blue drop. **c** Mean running speed in the anticipation zone across days and learning phases, data are presented as mean values + SEM. Individual data points are shown as grey dots and data from the same animal are linked by gray lines. *n* = 6 mice. One-way RM ANOVA and post hoc Holm-Sidak tests were used for days 1, 4, and 7. Paired t-test was used for days 2, 3, 5, and 6. $*p < 0.05$, $**p < 0.01$, $***p < 0.001$. Exact *p* values can be found in Supplementary Dataset 1. Source data are provided as a Source Data file.

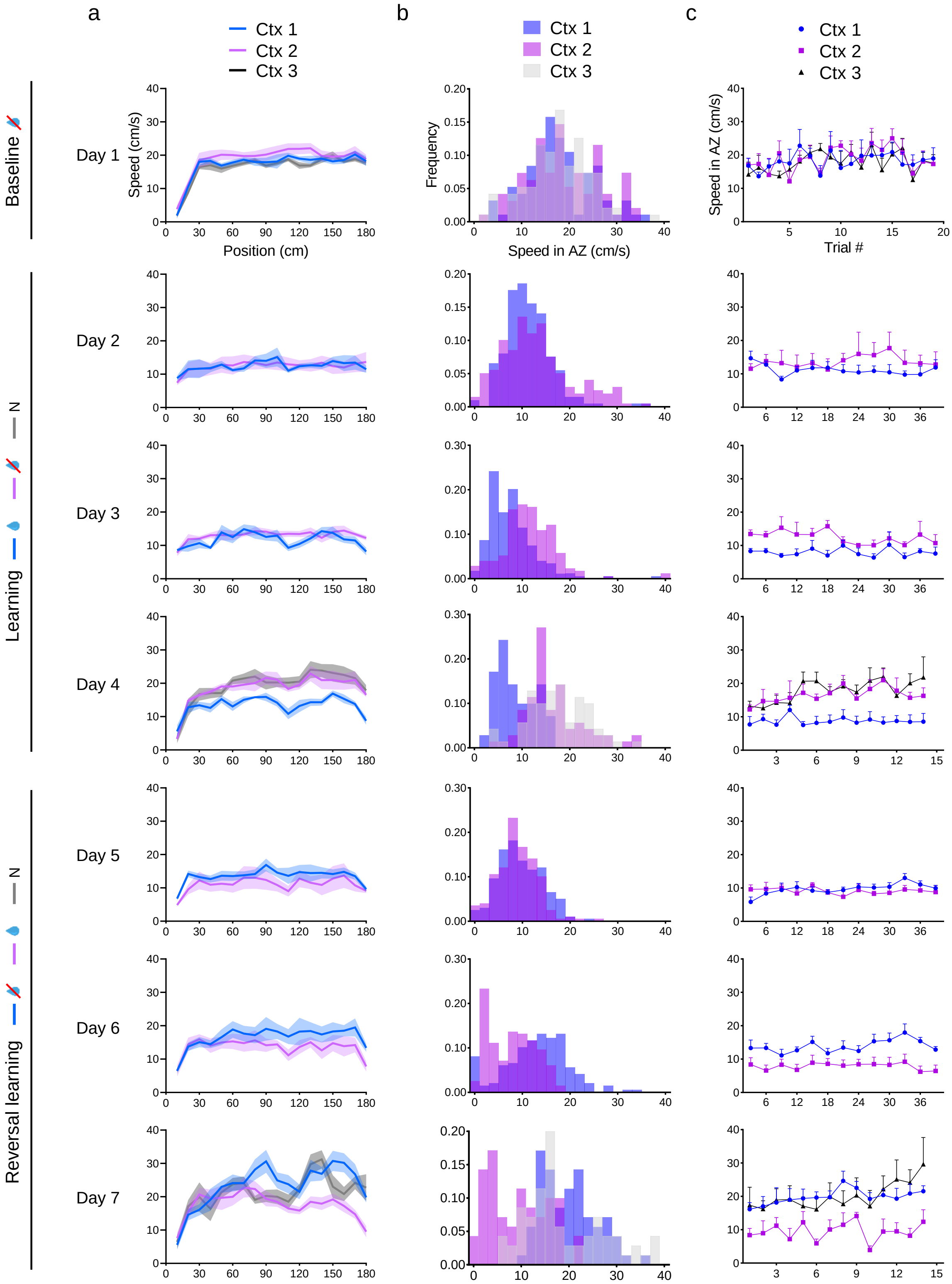

Supplementary Figure 2. Changes in speed along the length of the virtual corridor, and in the anticipation zone on a trial-by-trial basis both across and within sessions, during learning and reversal learning. Rewarded context (blue drop), non-rewarded context (drop with red cross), and neutral context (N). **a** Speed trajectories averaged across all animals in the control group, shown for each context and across each day. Solid lines indicate the mean speed across trials and shading indicates the corresponding SEM. **b** Distribution of speeds within the anticipation zone on a trial-by-trial basis across each day for the control group. **c** Within session trial-by-trial mean speed in the anticipation zone for each context, averaged across animals. For days with only two context presentations (day 2, 3, 5 and 6), data was binned across three trials. For all, *n* = 5 mice. For **a** and **c**, data are presented as mean values + SEM. Source data are provided as a Source Data file.

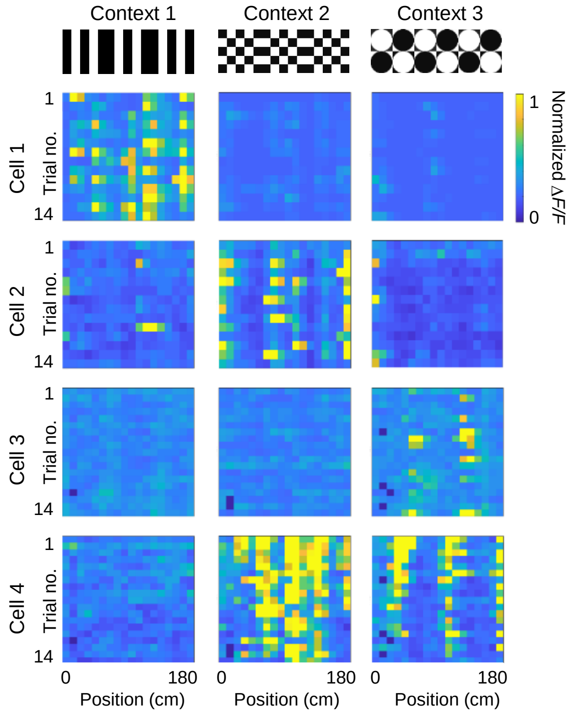

Supplementary Figure 3. Normalized responses (Δ*F/F*) along the virtual corridor (0-180 cm) for four example context-encoding neurons in the RSC; responses across multiple trials for each context are shown.

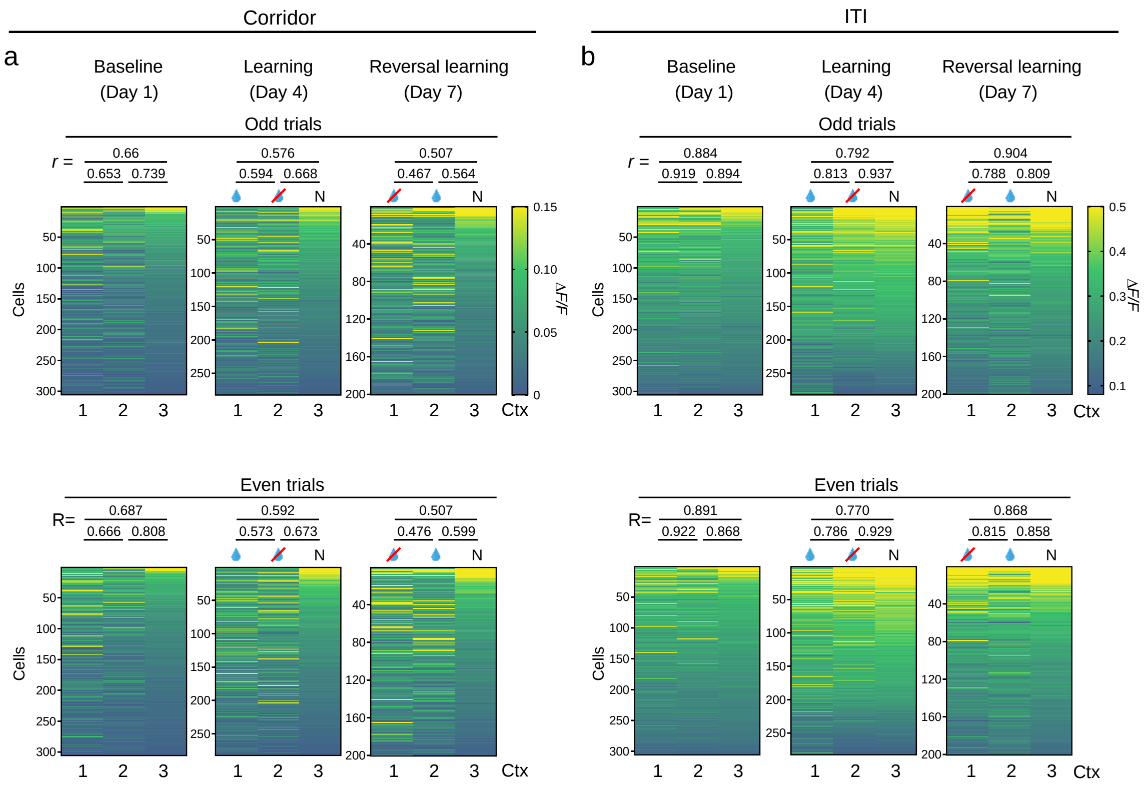

Supplementary Figure 4. Cross-validation of the similarity of neuronal activity across contexts in each learning phases. **a** Mean Δ*F/F* for each neuron averaged along the length of the virtual corridor for each context (Ctx 1-3) and across learning phases (day 1, 4, and 7) in odd and even trials, respectively. Cross-correlations (Spearman *r* value) of neuronal activity between contexts are reported on top of the color maps. **b** Same as **a** but during ITI. Source data are provided as a Source Data file.

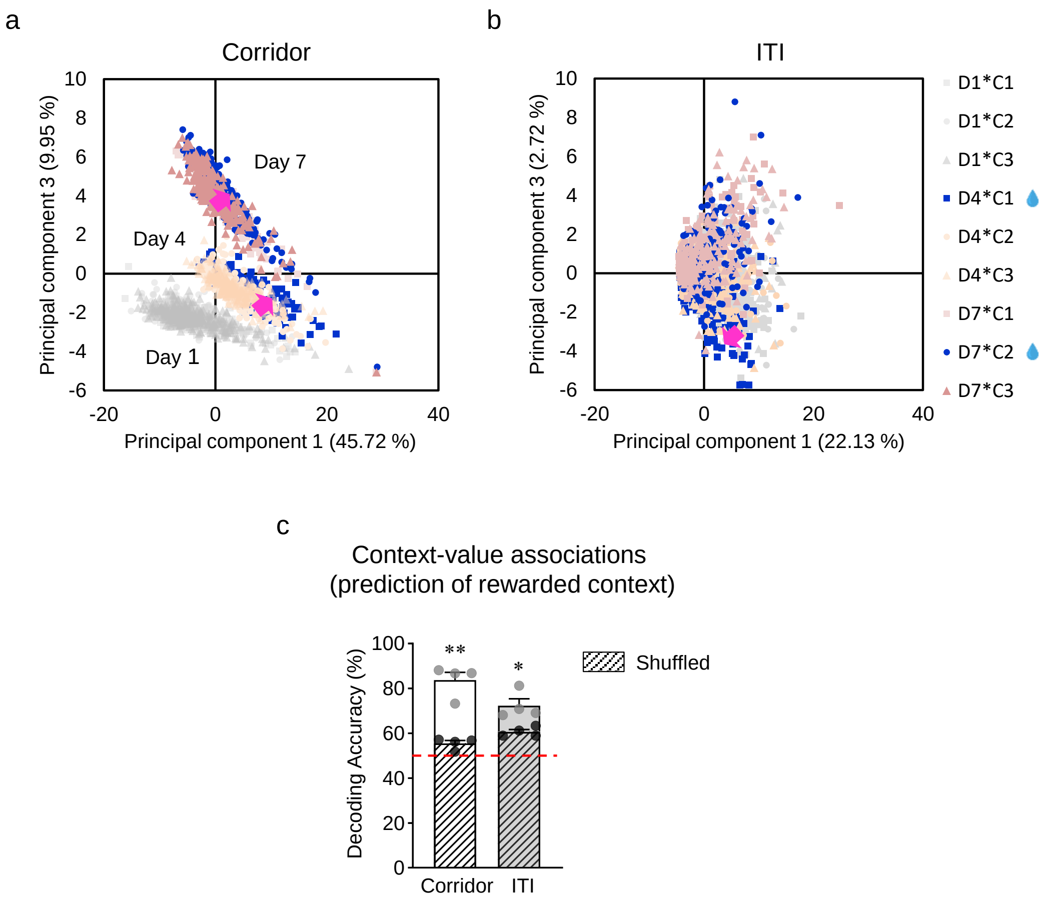

Supplementary Figure 5. Relationship between neuronal activity and context-value associations across contexts (C1, C2, C3) and days (D1, D4, D7). **a** Principal component analysis revealed a difference in representations of neuronal activity on different days and in contexts with different associated values (rewarded day-context pairs indicated by blue drop next to legend). Each point represents activity of 79 recorded cells per one representative animal at one space bin in one trial. Numbers in parenthesis correspond to % of variance explained by principal components. **b** same as in **a** except for the 3-second ITI period. **c** Linear discriminant analysis was used across all days and contexts to assess the prediction of the rewarded context-day associations (i.e. D4*C1 and D7*C2 combined versus all other combinations). Red dashed line represents chance level (rewarded vs non-rewarded). Bar plot represents the mean + SEM values for 4 mice. For **c**, one-sample t-test was used to compare to the hypothesized population mean (50 %), $*p < 0.05$, $**p < 0.01$. Exact p values can be found in Supplementary Dataset 1. Source data are provided as a Source Data file.

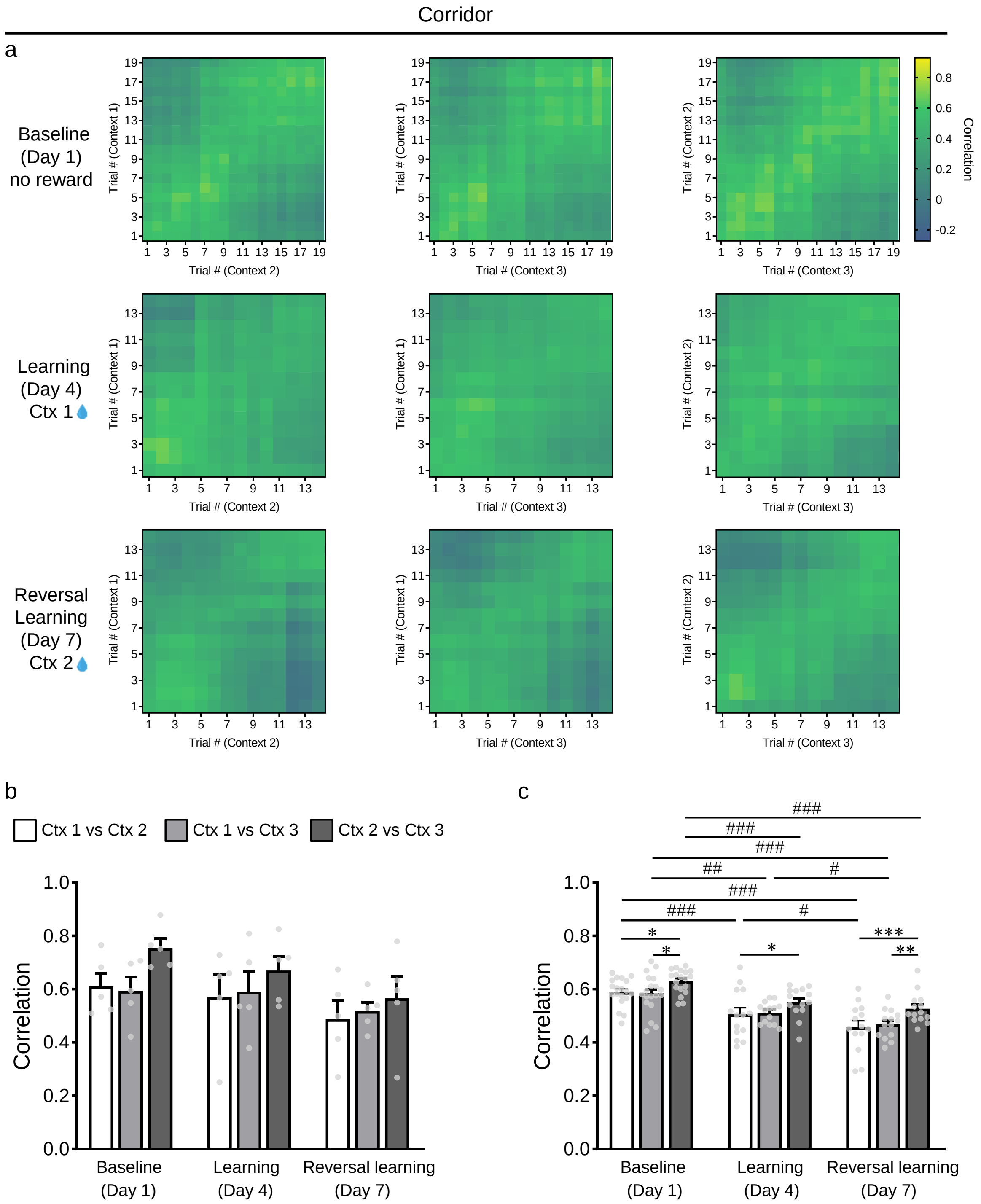

Supplementary Figure 6. Correlation of neuronal activity along the corridor across contexts in each learning phase. **a** Spearman correlation matrix between contexts (Ctx 1 vs Ctx 2, Ctx 1 vs Ctx 3, and Ctx 2 vs Ctx 3) across trials in each learning phase. Rewarded context for that learning phase indicated by blue drop. **b** Mean Spearman coefficients of correlation of neuronal activity between contexts across animals in each learning phase, n=5 mice. **c** Mean Spearman coefficients of correlation across trials along the main diagonal in **a**, $n = 19$ trials for Day 1 and $n = 14$ trials for Day 4 and 7. For **b** and **c**, data are presented as mean values + SEM, two-way RM ANOVA and post hoc Holm-Sidak tests were used. Within-day comparisons: $^{*}p < 0.05$, $^{**}p < 0.01$, $^{***}p < 0.001$; between-day comparisons: $^{\#}p < 0.05$, $^{\#\#}p < 0.01$, $^{\#\#\#}p < 0.001$. Exact p values can be found in Supplementary Dataset 1. Source data are provided as a Source Data file.

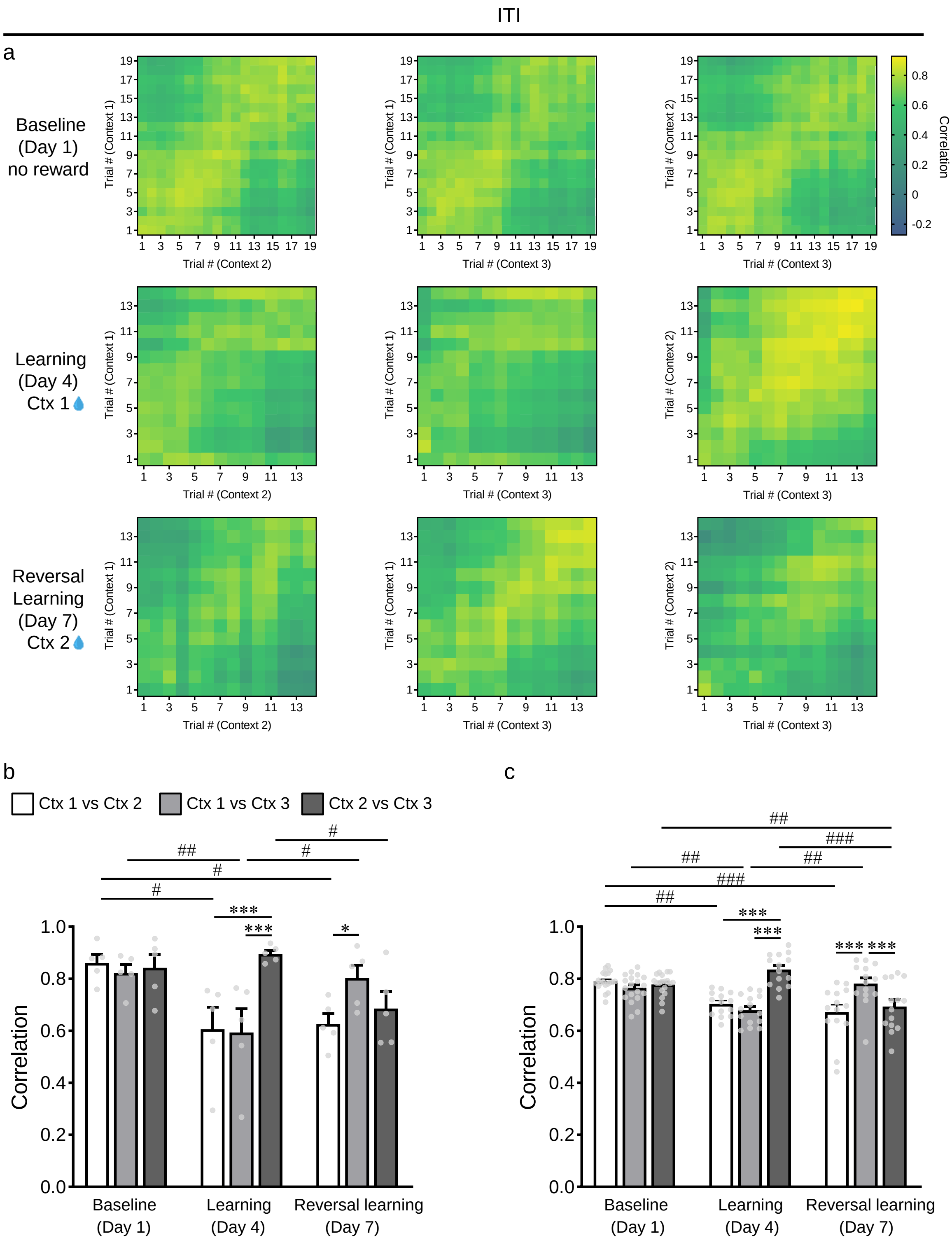

Supplementary Figure 7. Correlation of neuronal activity during the 3-second intertrial interval (ITI) across contexts in each learning phase. **a** Spearman correlation matrix between contexts (Ctx 1 vs Ctx 2, Ctx 1 vs Ctx 3, and Ctx 2 vs Ctx 3) across trials in each learning phase. Rewarded context for that learning phase indicated by blue drop. **b** Mean Spearman coefficients of correlation of neuronal activity between contexts across animals in each learning phase, n=5 mice. **c** Mean Spearman coefficients of correlation across trials along the main diagonal in **a**, n = 19 trials for Day 1 and n = 14 trials for Day 4 and 7. For **b** and **c**, data are presented as mean values + SEM, two-way RM ANOVA and post hoc Holm-Sidak tests were used. Within-day comparisons: $^{*}p < 0.05$, $^{***}p < 0.001$; between-day comparisons: $^{\#}p < 0.05$, $^{\#\#}p < 0.01$, $^{\#\#\#}p < 0.001$. Exact p values can be found in Supplementary Dataset 1. Source data are provided as a Source Data file.

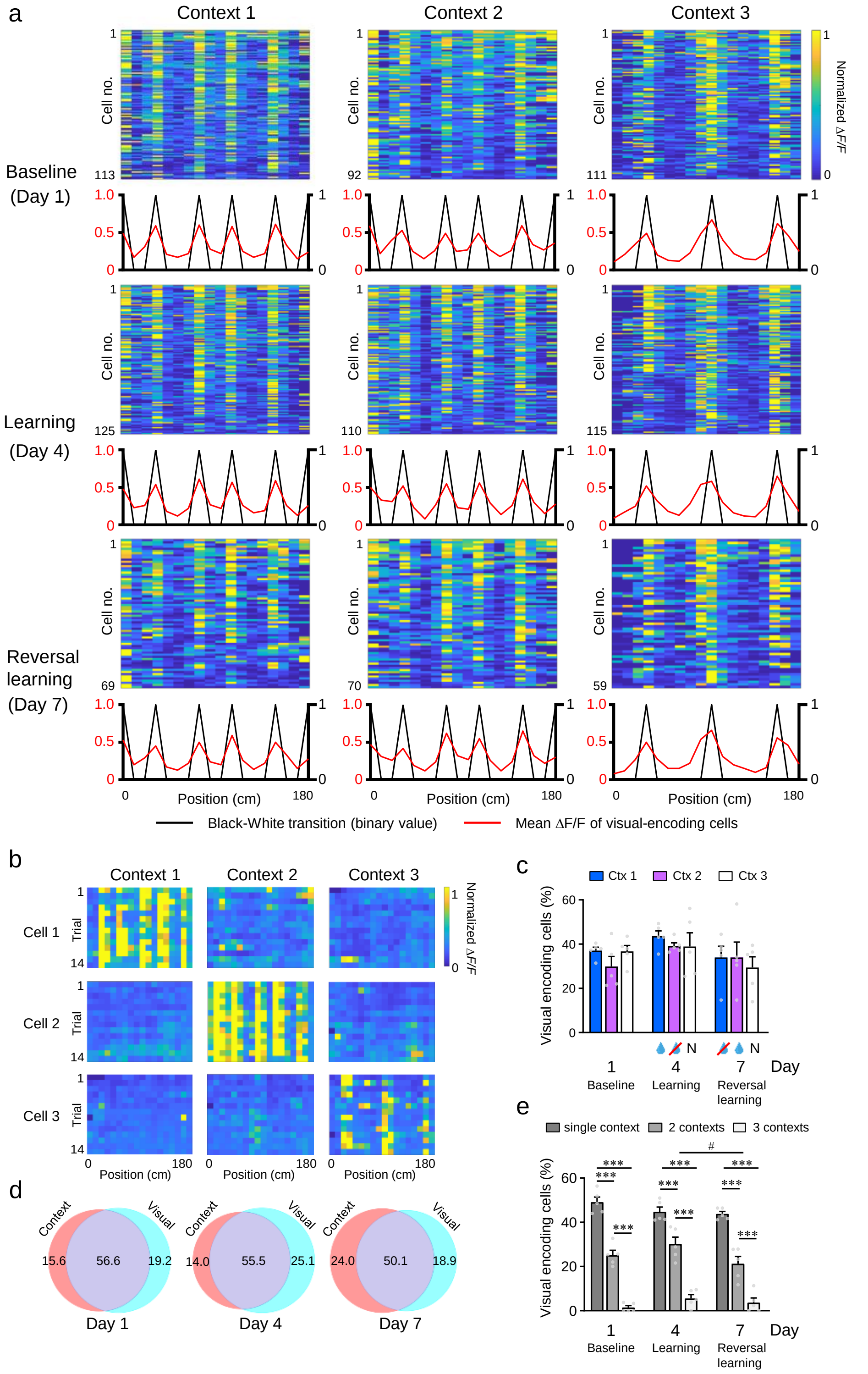

Supplementary Figure 8. Neurons in the RSC respond to the visual properties of the virtual environment. **a** Correlation between the responses (Δ*F/F*) of visual encoding cells and visual properties of the virtual environment (dark-light transitions). Heatmaps show the normalized Δ*F/F* of all visual encoding cells from 5 mice, aligned by the time lag calculated using Pearson cross-correlation. Line graphs show the mean Δ*F/F* of all visual encoding cells (red line) and dark-light transitions of the stimulus (black line). **b** Normalized responses (Δ*F/F*) along the virtual corridor (0-180 cm) for three visual-encoding neurons in the RSC; responses across multiple trials for each context. **c** Proportion of visual encoding cells across contexts and days. **d** Percentage of neurons encoding context and visual properties on each day. **e** Context specificity of visual encoding cells on each day. For **c, d,** and **e**, n = 5 mice. For **c** and **e**, data are presented as mean values + SEM. Two-way RM ANOVA and post hoc Holm-Sidak tests were used. Within-day comparisons: ***$p < 0.001$; between-day comparisons: $^{\#}p < 0.05$. Exact p values can be found in Supplementary Dataset 1. Source data are provided as a Source Data file.

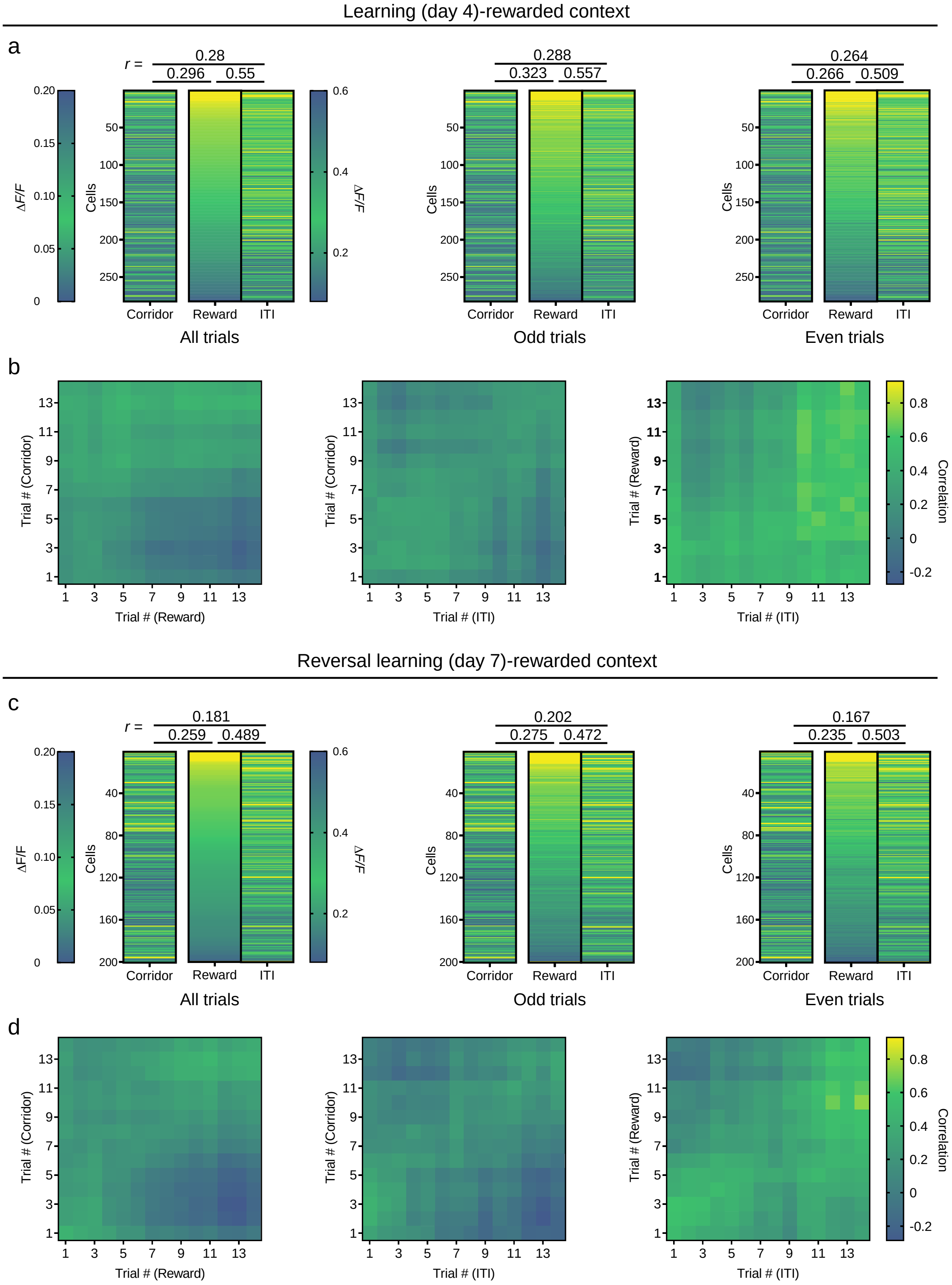

Supplementary Figure 9. Neuronal responses in the rewarded context across the corridor, reward period, and intertrial interval (ITI) across learning and reversal learning phases. **a** Mean Δ*F/F* in the rewarded context for each neuron averaged along the length of the virtual corridor, during the delivery of the water reward, and during the following 3-second ITI for learning phase (day 4). Cells are sorted according to the mean Δ*F/F* during the reward period for all data and sorting order is kept consistent for odd and even trials. The scale on the left applies to the virtual corridor and the scale on the right applies to both reward and ITI panels. Cross-validation using odd and even trials are shown, respectively. Cross-correlations (Spearman *r* value) of neuronal activity between columns are reported on top of the color maps. **b** Spearman correlation matrix of neuronal responses between corridor, reward period, and ITI across all trials in learning phase (day 4). **c** Same as **a** but within reversal learning phase (day 7). **d** Same as **b** but within reversal learning phase (day 7). Source data are provided as a Source Data file.

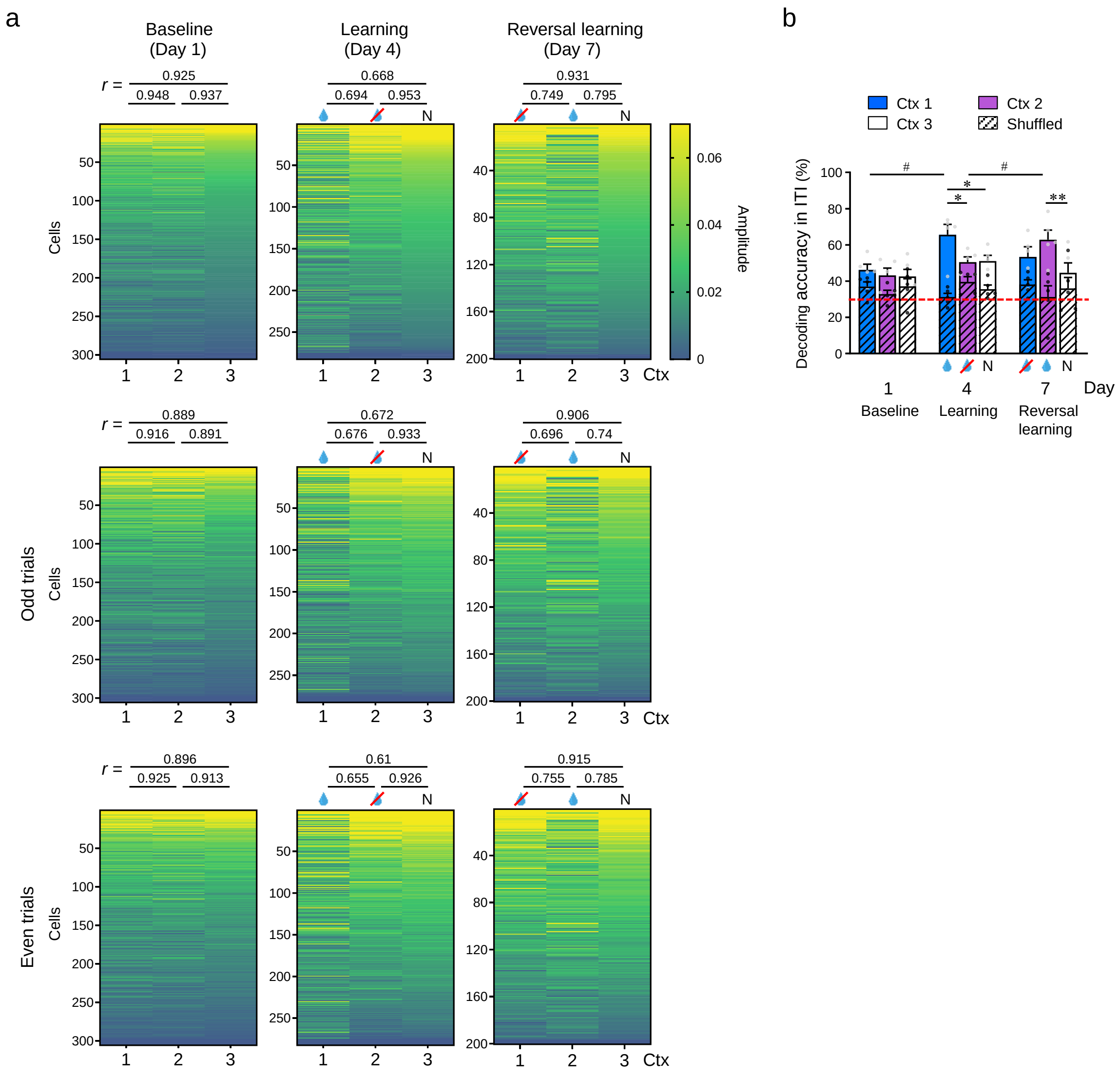

Supplementary Figure 10. Neuronal responses and decoding accuracy within the intertrial interval (ITI) after deconvolution. **a** Mean amplitude of the deconvolved spike inference signal for each neuron averaged in the ITI for each context (1-3) and across learning phases (day 1, 4, and 7). Cells are sorted for each day according to the mean amplitude for the neutral (N) context. Cross-validation plots using odd and even trials are shown below, respectively. For all, cross-correlations (Spearman r value) of neuronal activity between contexts are reported on top of the color maps. **b** Decoding accuracy for each context across learning phases. Results after random shuffling of the raw data (hatched pattern) and red dashed line indicating the chance level (33.3%) are shown. Data are presented as mean values + SEM. Two-way RM ANOVA and post hoc Holm-Sidak tests were used. Within-day comparisons: *$p < 0.05$, **$p < 0.01$; between-day comparisons: $^{\#}p < 0.05$, $n = 5$ mice. Exact p values can be found in Supplementary Dataset 1. Source data are provided as a Source Data file.

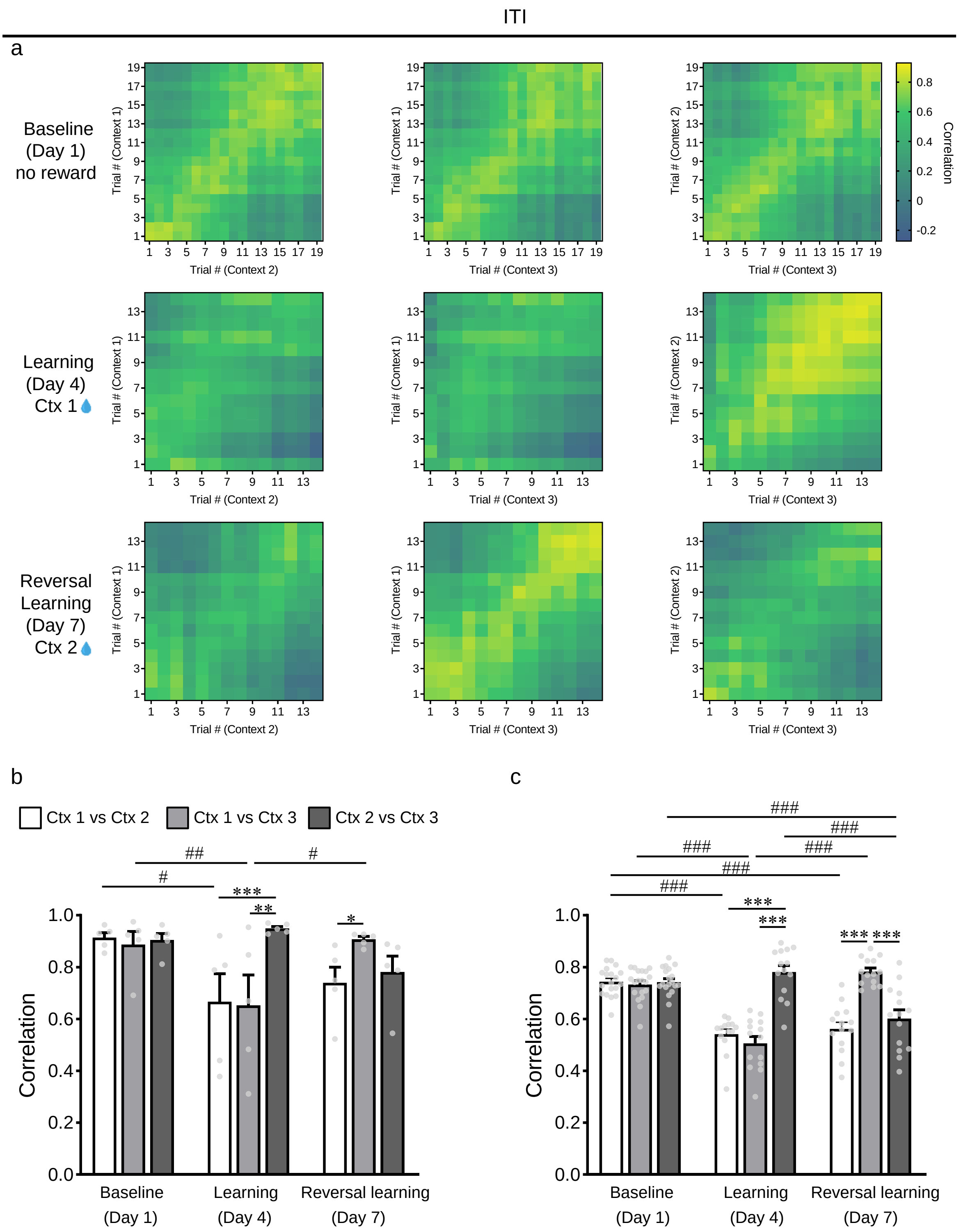

Supplementary Figure 11. Correlation of neuronal activity (amplitude after deconvolution) during the 3-second intertrial interval (ITI) period across contexts in each learning phase. **a** Spearman correlation matrix between contexts (Ctx 1 vs Ctx 2, Ctx 1 vs Ctx 3, and Ctx 2 vs Ctx 3) across trials in each learning phase. Rewarded context for that learning phase indicated by blue drop. **b** Mean Spearman correlation of neuronal activity between contexts across animals in each learning phase, n=5 mice. **c** Mean Spearman correlation across trials along the main diagonal in **a**, $n = 19$ trials for Day 1 and $n = 14$ trials for Day 4 and 7. For **b** and **c**, data are presented as mean values + SEM, two-way RM ANOVA and post hoc Holm-Sidak tests were used. Within-day comparisons: $*p < 0.05$, $**p < 0.01$, $***p < 0.001$; between-day comparisons: $^{\#}p < 0.05$, $^{\#\#}p < 0.01$, $^{\#\#\#}p < 0.001$. Exact p values can be found in Supplementary Dataset 1. Source data are provided as a Source Data file.

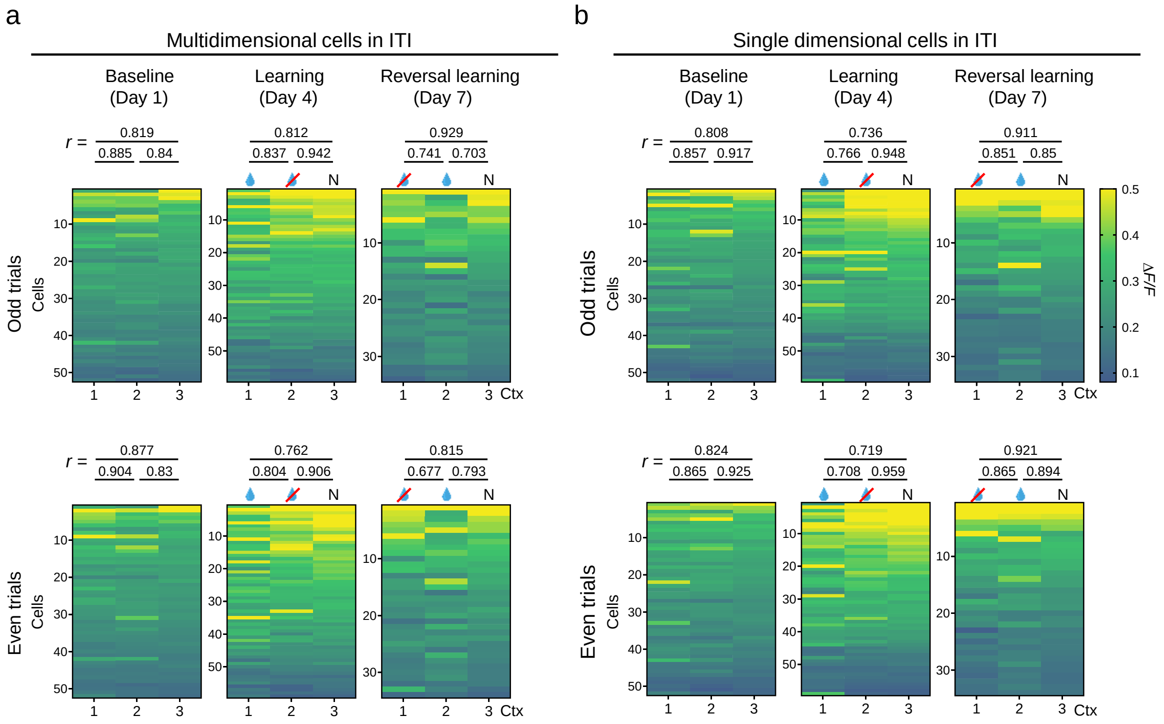

Supplementary Figure 12. Cross-validation of the multidimensional and single-dimensional neuronal activity across contexts (Ctx 1-3) and learning phases. **a** Mean Δ*F/F* for each multidimensional neuron averaged during the intertrial interval (ITI) for each context (1-3) and across learning phases (day 1-baseline, 4-learning, and 7-reversal learning) in odd and even trials, respectively. Cross-correlations (Spearman *r* value) of neuronal activity between contexts are reported on top of the color maps. **b** Same as **a** but for single-dimensional cells. Source data are provided as a Source Data file.

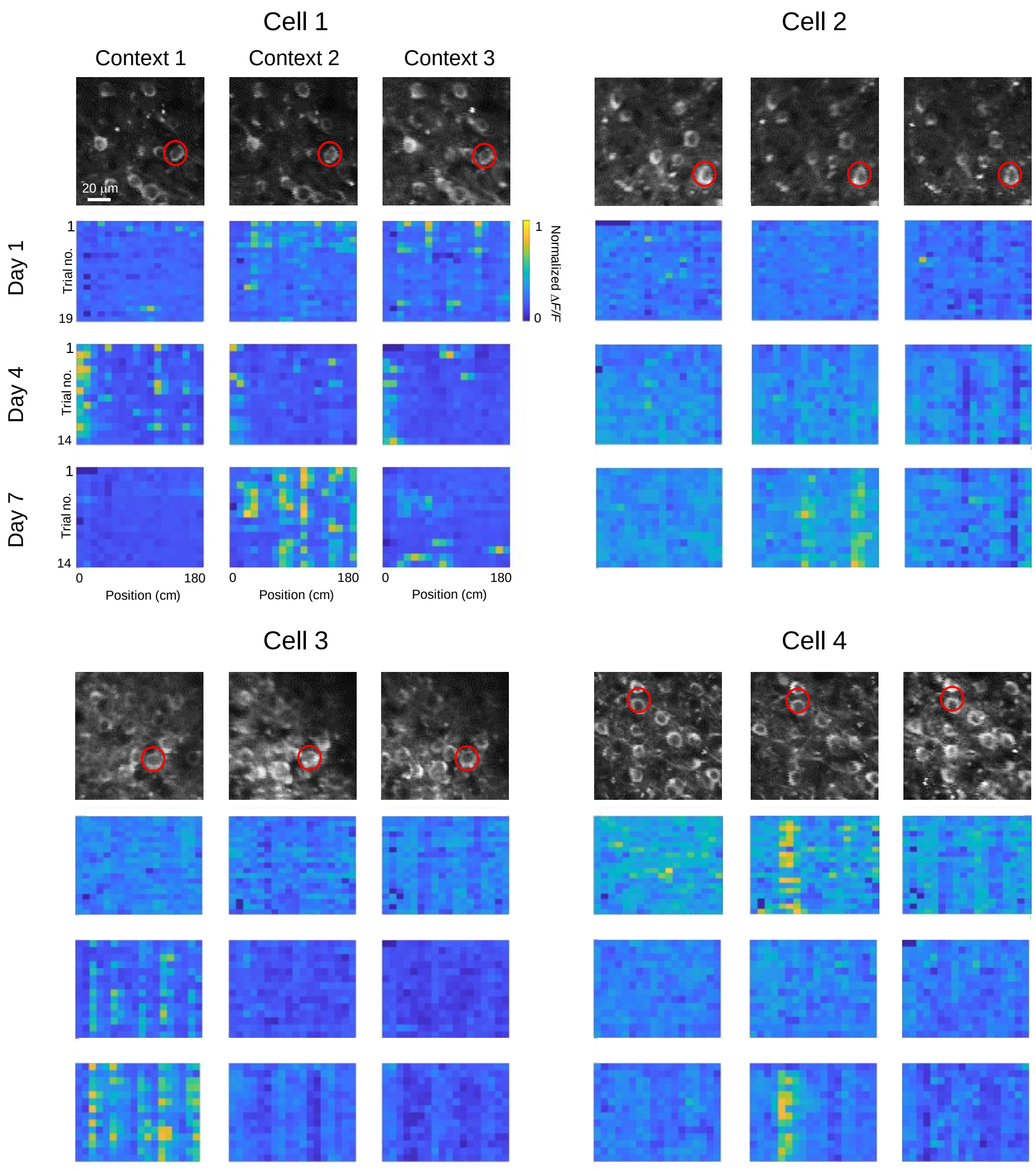

Supplementary Figure 13. Chronic imaging of the same neurons across days. Normalized responses ($\Delta F/F$) along the virtual corridor (0-180 cm) of exemplary RSC neurons (red circle) across days. Responses are shown across trials for each context and for baseline (day 1) as well as after learning (day 4) and after reversal learning (day 7). This experiment was repeated independently in 4 mice and 3 sessions for each mouse.

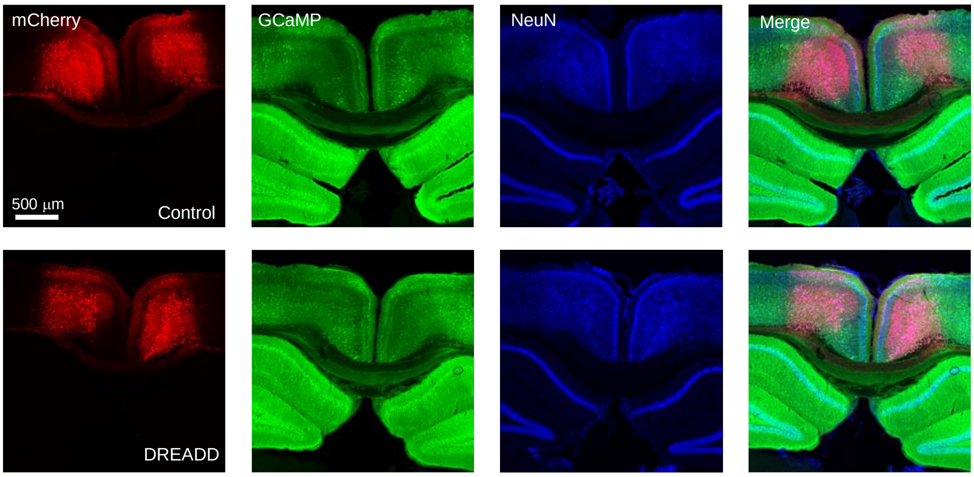

Supplementary Figure 14. Control AAV and DREADD expression in RSC. Either a control AAV (AAV8/hSyn-mCherry) driving mCherry expression (top) or an AAV (AAV8/hSyn-hM4Di-mCherry) driving DREADD and mCherry expression (bottom) was expressed throughout the RSC. NeuN (blue), GCaMP6f (green), mCherry (red). This experiment was repeated independently in 5 mice for the control group and 7 mice for the DREADD group with similar results.

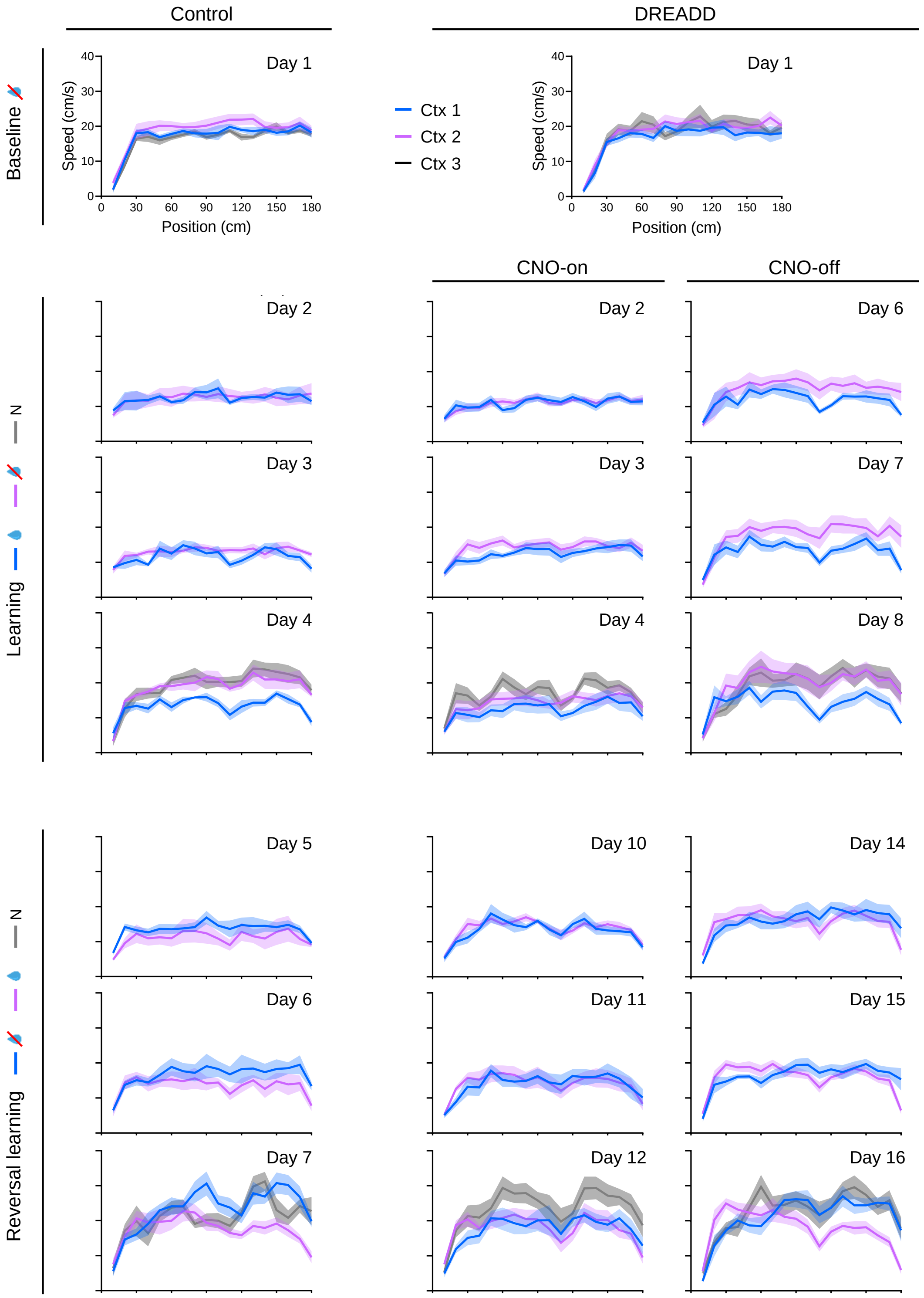

Supplementary Figure 15. Speed trajectories averaged across all animals in the control group, shown for each context (Ctx 1=3) and across each day. Solid lines indicate the mean speed and shading indicates the corresponding SEM. Data are shown for the control group (left) with a control AAV expressing mCherry injected and CNO applied for day 2-7 (*n* = 5 mice) and for a DREADD experimental group with injection of AAV8/hSyn-hM4Di-mCherry into the RSC (*n* = 7 mice) and with CNO indicated as on (middle) or off (right) depending on the experimental day. Source data are provided as a Source Data file.

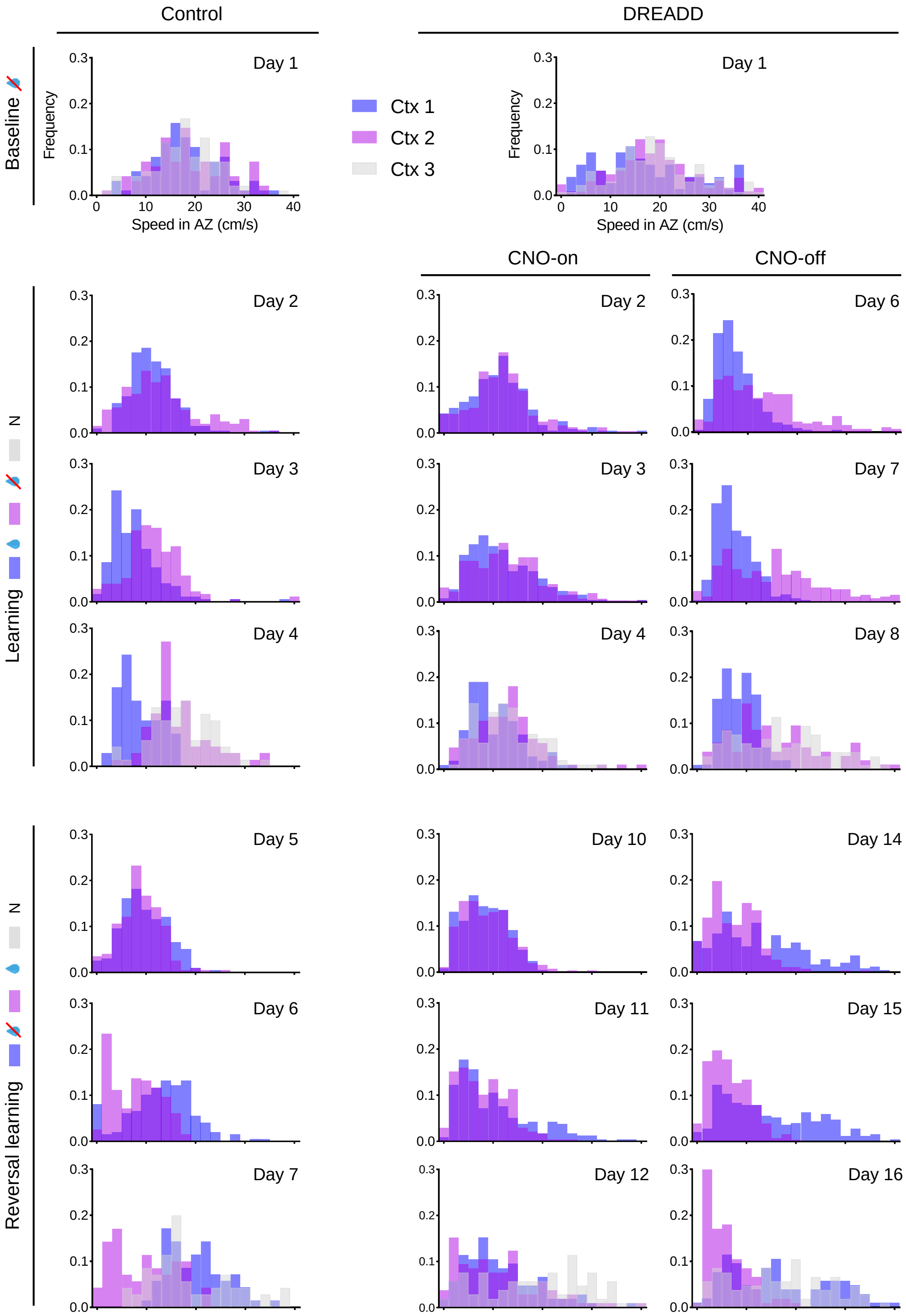

Supplementary Figure 16. Distribution of running speeds within the anticipation zone on a trial-by-trial basis across all days. Data are shown for the control group (left) with a control AAV expressing mCherry injected and CNO applied for day 2-7 (n = 5 mice) and for a DREADD experimental group with injection of AAV8/hSyn-hM4Di-mCherry into the RSC (n = 7 mice) and with CNO indicated as on (middle) or off (right) depending on the experimental day. Source data are provided as a Source Data file.

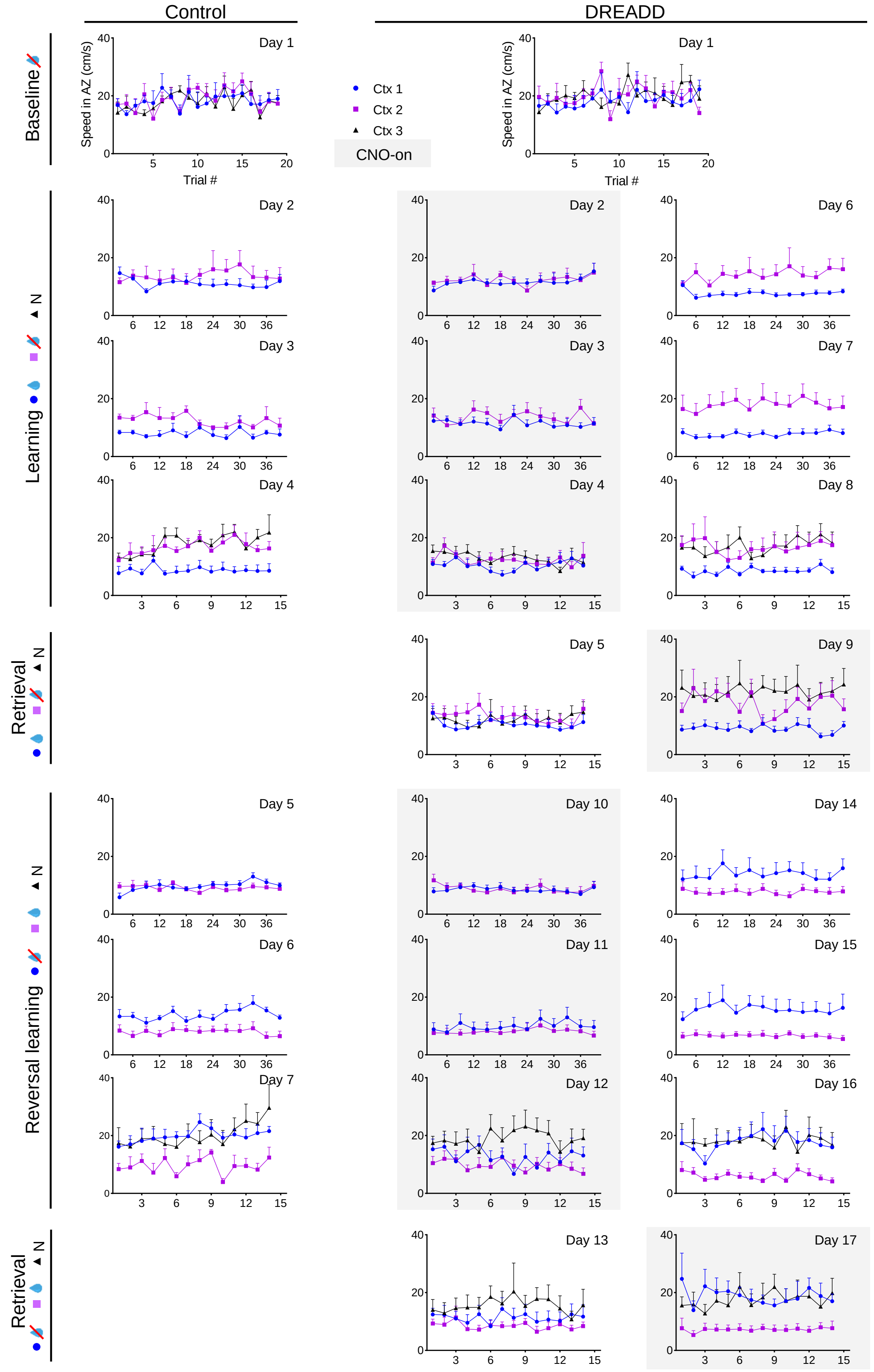

Supplementary Figure 17. Within session trial-by-trial mean speed in the anticipation zone for each context, averaged across animals. For days with only two context presentations (e.g. days 2, 3, 5 and 6), data was binned across three trials. Data are presented as mean values + SEM. Data are shown for the control group (left) with a control AAV expressing mCherry injected and CNO applied for day 2-7 (*n* = 5 mice) and for a DREADD experimental group with injection of AAV8/hSyn-hM4Di-mCherry into the RSC (*n* = 7 mice) and with CNO indicated as on (grey background) or off (no background) depending on the experimental day. Source data are provided as a Source Data file.

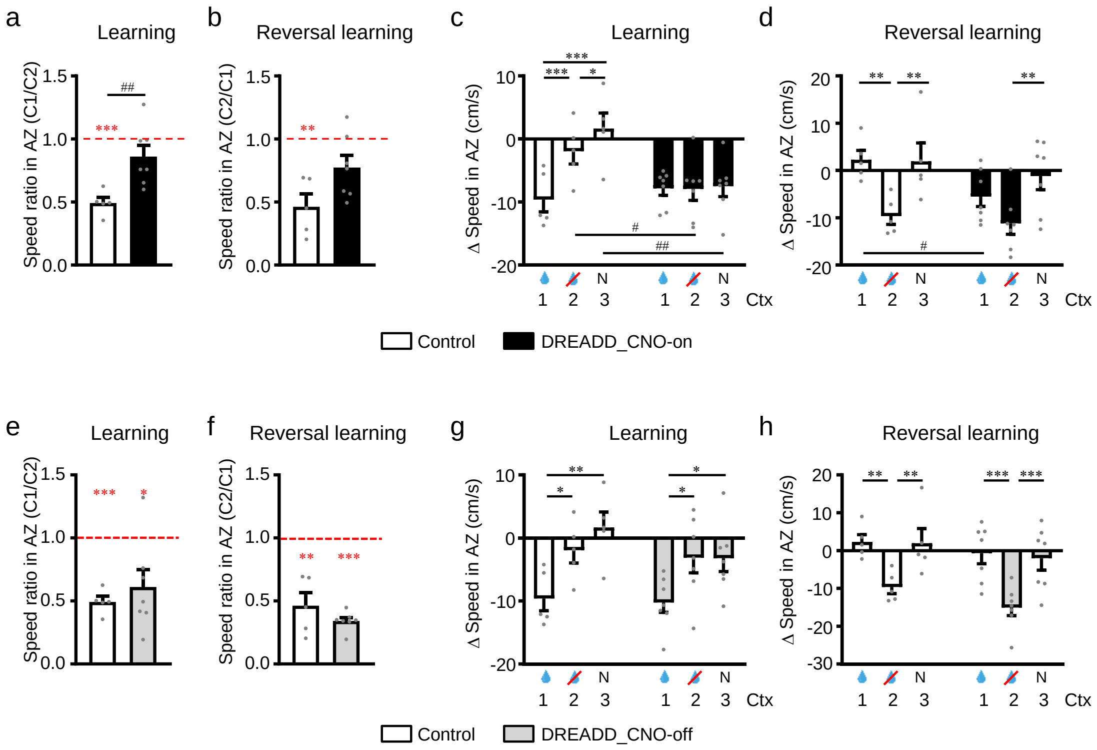

Supplementary Figure 18. Comparison of running speed between control and DREADD group with CNO-on or CNO-off. **a** Ratio of speed in the anticipation zone between rewarded context (C1) and non-rewarded context (C2) after learning (day 4). **b** Ratio of speed in the anticipation zone between rewarded context (C2) and non-rewarded context (C1) after reversal learning (control group: day 7 and DREADD group: day 12). **c** Change in speed relative to baseline for learning (day *i*-day 1) and reversal learning (day *i*-day 1). Rewarded context (blue drop), non-rewarded context (drop with red cross), and neutral context (N). Data shown for the control group (day 4) and DREADD group with CNO-on (day 4). **d** Same as **c** but after reversal learning (control: day 7-day 1; DREADD CNO-on: day 12-day 1). **e** Same as **a** but CNO-off for DREADD (day 8). **f** Same as **b** but CNO-off for DREADD (day 16). **g** Same as **c** but CNO-off for DREADD (day 8). **h** Same as **d** but CNO-off for DREADD (day 16). For all, data are presented as mean values + SEM. For **a**, **b**, **e**, and **f**, paired t-test was used for comparisons between groups and one-sample t-test was used to compare to the hypothesized population mean (1.00). Within-day comparisons: *$p < 0.05$, **$p < 0.01$, ***$p < 0.001$; between-day comparisons: $^{\#\#}p < 0.01$. For control group, $n = 5$ mice and for DREADD group, $n = 7$ mice. Exact p values can be found in Supplementary Dataset 1. Source data are provided as a Source Data file.
