# Supplementary material for: Context value updating and multidimensional neuronal encoding in the retrosplenial cortex
Source: Nat Commun. 2021 Oct 18;12:6045. doi: 10.1038/s41467-021-26301-z (PMC8523535; doi:10.1038/s41467-021-26301-z)
Supplement: Supplementary file 3 — Description of Additional Supplementary Files [file 41467_2021_26301_MOESM3_ESM.pdf]

Description of Additional Supplementary Files

Supplementary Data 1: A set of datasheets containing information about the statistical test used, F- or t-values, degrees of freedom, and p-values for all statistical analyses presented in main and supplementary figures.
