# Supplementary material for: Context value updating and multidimensional neuronal encoding in the retrosplenial cortex
Source: Nat Commun. 2021 Oct 18;12:6045. doi: 10.1038/s41467-021-26301-z (PMC8523535; doi:10.1038/s41467-021-26301-z)
Supplement: Supplementary file 5 — Reporting summary [file 41467_2021_26301_MOESM5_ESM.pdf]

nature research

Corresponding author(s): Janelle Pakan and Alexander DItyatev

Last updated by author(s): Sep 15, 2021

Reporting Summary

Nature Research wishes to improve the reproducibility of the work that we publish. This form provides structure for consistency and transparency in reporting. For further information on Nature Research policies, see our Editorial Policies and the Editorial Policy Checklist.

Statistics

For all statistical analyses, confirm that the following items are present in the figure legend, table legend, main text, or Methods section.

n/a	Confirmed	
☐	☒	The exact sample size (*n*) for each experimental group/condition, given as a discrete number and unit of measurement
☐	☒	A statement on whether measurements were taken from distinct samples or whether the same sample was measured repeatedly
☐	☒	The statistical test(s) used AND whether they are one- or two-sided<br>*Only common tests should be described solely by name; describe more complex techniques in the Methods section.*
☐	☒	A description of all covariates tested
☐	☒	A description of any assumptions or corrections, such as tests of normality and adjustment for multiple comparisons
☐	☒	A full description of the statistical parameters including central tendency (e.g. means) or other basic estimates (e.g. regression coefficient) AND variation (e.g. standard deviation) or associated estimates of uncertainty (e.g. confidence intervals)
☐	☒	For null hypothesis testing, the test statistic (e.g. *F*, *t*, *r*) with confidence intervals, effect sizes, degrees of freedom and *P* value noted<br>*Give P values as exact values whenever suitable.*
☒	☐	For Bayesian analysis, information on the choice of priors and Markov chain Monte Carlo settings
☒	☐	For hierarchical and complex designs, identification of the appropriate level for tests and full reporting of outcomes
☐	☒	Estimates of effect sizes (e.g. Cohen's *d*, Pearson's *r*), indicating how they were calculated

Our web collection on statistics for biologists contains articles on many of the points above.

Software and code

Policy information about availability of computer code

Data collection	ThorImageLS imaging software (version 2.4, THORLABS). PhenoSoft VR (version 1.0.6717.25007, PhenoSys). ZEN 3.0 SR black edition (version 3.0.0.0, Carl Zeiss). Imaging and behavioral data were synchronized by custom-written code (MATLAB, MathWorks), which is available on GitHub (https://github.com/seobseob/MATLAB-code).
Data analysis	We used the open-source motion-correction program NoRMCorre (version 0.1.1, Pnevmatikakis et al., 2017), automatic ROI detection software suite2p (version 0.7.1, Pachitariu et al., 2016) and CalmAn-MATLAB (cNMF method including deconvolution; version 0.7, Pnevmatikakis et al., 2016). For further analysis and plotting, XLSTAT (version 23.3.1150, Basic+, Addinsoft), GraphPad Prism (version 8.2.1, GraphPad Software) and SigmaPlot (version 13; Systat Software) were used.

For manuscripts utilizing custom algorithms or software that are central to the research but not yet described in published literature, software must be made available to editors and reviewers. We strongly encourage code deposition in a community repository (e.g. GitHub). See the Nature Research guidelines for submitting code & software for further information.

Data

Policy information about availability of data

All manuscripts must include a data availability statement. This statement should provide the following information, where applicable:

- Accession codes, unique identifiers, or web links for publicly available datasets
- A list of figures that have associated raw data
- A description of any restrictions on data availability

Data availability: The source data underlying all figures are provided as a Source Data file. The raw ΔF/F ΔF/F and speed values at each position bin along the virtual corridor and at each time bin during ITI data used in this study are publicly available in the Mendeley Data database with https://data.mendeley.com/datasets/cpgrd49h85/1. All two-photon images are publicly available in the Mendeley Data database with https://data.mendeley.com/datasets/dcv885kzf2/1, https://

data.mendeley.com/datasets/k9z74vfgwf/1, and https://data.mendeley.com/datasets/hjgtsmyyv5/1. Source data are provided with this paper.
Code availability: The code used to synchronize two-photon imaging with behavioral data, and to calculate the speed from Phenosys data are available on GitHub (https://github.com/seobseob/MATLAB-code).

Field-specific reporting

Please select the one below that is the best fit for your research. If you are not sure, read the appropriate sections before making your selection.

- [x] Life sciences
- [ ] Behavioural & social sciences
- [ ] Ecological, evolutionary & environmental sciences

For a reference copy of the document with all sections, see nature.com/documents/nr-reporting-summary-flat.pdf

Life sciences study design

All studies must disclose on these points even when the disclosure is negative.

Sample size	Sample size was selected based on power analysis which shows that 5 mice per group provides sufficient statistical power and also according to previous studies (see refs 20, 24, 28, 76), in which 5-7 mice per group were used in the similar experiments. Therefore, experiments were performed with 5-7 mice per group in this study.
Data exclusions	One of five mice was excluded from remapping analysis (Figure 5d) due to the field-of-view on baseline day not precisely matching to the following imaging days.
Replication	Behavioral results, measured by running speed, were replicated across days for three separate groups of animals with either no AAV injection, a control AAV injection in RSC driving the expression of mCherry, or an AAV driving the expression of mCherry and a inhibitory DREADDs into the RSC. Two open source pipelines for automatic ROI detection and signal analysis - suite2p (Pachitariu et al., 2016) and CalmAn-MATLAB (including deconvolution; Pnevmatikakis et al., 2016) methods - have been used and provided similar results in terms of accuracy of context, position, and speed encoding.
Randomization	Mice were randomly assigned to groups which received injection of control (AAV8/hSyn-mCherry) or inhibitory DREADD (AAV8/hSyn-hM4Di-mCherry) AAVs using generic ID numbers.
Blinding	As mice in the AAV8/hSyn-hM4Di-mCherry group had an altered experimental timeline with CNO-on and CNO-off, compared to the AAV8/hSyn-mCherry group, it was impossible to blind the group identity.

Reporting for specific materials, systems and methods

We require information from authors about some types of materials, experimental systems and methods used in many studies. Here, indicate whether each material, system or method listed is relevant to your study. If you are not sure if a list item applies to your research, read the appropriate section before selecting a response.

Materials & experimental systems

n/a	Involved in the study	
[ ]	[x]	Antibodies
[x]	[ ]	Eukaryotic cell lines
[x]	[ ]	Palaeontology and archaeology
[ ]	[x]	Animals and other organisms
[x]	[ ]	Human research participants
[x]	[ ]	Clinical data
[x]	[ ]	Dual use research of concern

Methods

n/a	Involved in the study	
[x]	[ ]	ChIP-seq
[x]	[ ]	Flow cytometry
[x]	[ ]	MRI-based neuroimaging

Antibodies

Antibodies used	Primary antibodies: chicken anti-GFP, 1:500, abcam, ab13970, RRID: AB_300798; goat anti-mCherry, 1:200, SICGEN, AB0040-200, RRID: AB_2333092; mouse anti-NeuN, 1:500, Merck Millipore, MAB377, RRID: AB_2298772. Secondary antibodies: Alexa Fluor 488 donkey anti-chicken, 1:500, Jackson ImmunoResearch, 703-545-155, RRID: AB_2340375; Alexa Fluor 568 donkey anti-goat, 1:500, abcam, ab175704, RRID: AB_2725786; Alexa Fluor 647 donkey anti-mouse, 1:500, ThermoFisher, A31571, RRID: AB_162542.
Validation	All primary antibodies are commercially available and referenced by multiple studies. The antibodies were validated by suppliers as stated in the product information datasheet. Validation statement for each primary antibody is provided on the manufacturer's website. For chicken anti-GFP (ab13970), it has been validated for use in WB, ICC/IF for the detection of GFP and referenced in 2506 publications (https://www.abcam.com/gfp-antibody-ab13970.html). For goat anti-mCherry (AB0040), it has been validated for the use of WB, IF, and IHC and referenced in 49 publications (http://www.sicgen.pt/product/mcherry-polyclonal-antibody_1_27). For mouse anti-NeuN (MAB377), it has been validated for use in ICC, IHC, IH(P), and WB for the detection of NeuN and referenced in 47 publications (https://www.merckmillipore.com/DE/en/product/Anti-NeuN-Antibody-clone-A60-biotin-conjugated,MM_NF-

MAB377B#overview).

Animals and other organisms

Policy information about studies involving animals; ARRIVE guidelines recommended for reporting animal research

Laboratory animals	Eighteen Thy1-GCaMP6f mice with C57BL/6J genetic background (C57BL/6J-Tg [Thy1-GCaMP6f] GP5.5Dkim/J, Jackson Laboratory, USA; RRID: IMSR_JAX: 024276) were used for experiments (5- to 6-month-old males).
Wild animals	No wild animals were used in this study.
Field-collected samples	No field-collected samples were used in this study.
Ethics oversight	All mice were cared and treated strictly following the ethical animal research standards defined by the Directive of the European Communities Parliament and Council on the protection of animals used for scientific purposes (2010/63/EU) and were approved by the Ethical Committee on Animal Health and Care of Saxony-Anhalt state, Germany (license number: 42502-2-1346).

Note that full information on the approval of the study protocol must also be provided in the manuscript.
